# Redox Homeostasis and Prospects for Therapeutic Targeting in Neurodegenerative Disorders

**DOI:** 10.1155/2021/9971885

**Published:** 2021-08-03

**Authors:** Musbau Adewumi Akanji, Damilare Emmanuel Rotimi, Tobiloba Christiana Elebiyo, Oluwakemi Josephine Awakan, Oluyomi Stephen Adeyemi

**Affiliations:** ^1^Department of Biochemistry, University of Ilorin, Ilorin, Nigeria; ^2^Landmark University SDG 3 (Good Health and Well-being Research Group), PMB 1001, Omu-Aran 251101, Nigeria; ^3^Department of Biochemistry, Medicinal Biochemistry, Nanomedicine & Toxicology Laboratory, Landmark University, KM 4 Ipetu Road, Omu-Aran 251101, Kwara State, Nigeria

## Abstract

Reactive species, such as those of oxygen, nitrogen, and sulfur, are considered part of normal cellular metabolism and play significant roles that can impact several signaling processes in ways that lead to either cellular sustenance, protection, or damage. Cellular redox processes involve a balance in the production of reactive species (RS) and their removal because redox imbalance may facilitate oxidative damage. Physiologically, redox homeostasis is essential for the maintenance of many cellular processes. RS may serve as signaling molecules or cause oxidative cellular damage depending on the delicate equilibrium between RS production and their efficient removal through the use of enzymatic or nonenzymatic cellular mechanisms. Moreover, accumulating evidence suggests that redox imbalance plays a significant role in the progression of several neurodegenerative diseases. For example, studies have shown that redox imbalance in the brain mediates neurodegeneration and alters normal cytoprotective responses to stress. Therefore, this review describes redox homeostasis in neurodegenerative diseases with a focus on Alzheimer's and Parkinson's disease. A clearer understanding of the redox-regulated processes in neurodegenerative disorders may afford opportunities for newer therapeutic strategies.

## 1. Introduction

Neurodegeneration, which is characterized by gradual neuronal and synaptic degradation, glial focal proliferation, neuroinflammation, vascular abnormalities in specific brain regions, and modifications of proteins such as *α*-synuclein, amyloid-*β*, and tau proteins [1–3], contributes significantly to the global health burden. The major groups of neurodegenerative diseases include Alzheimer's disease (AD), Parkinson's disease (PD), spinal muscular atrophy (SMA), prion diseases, spinocerebellar ataxia (SCA), motor neuron disease (MND), and Huntington's disease (HD). Different neurodegenerative diseases have a variety of pathophysiologies, which include cognitive and memory impairments or the inability to move, speak, or breathe [4].

In the brain, a concomitant low activity of antioxidant defense mechanisms and the presence of highly redox-active metals (copper and iron) allow for greater sensitivity to reactive oxygen species (ROS) than other organs [5]. Although oxygen is necessary for life, an imbalance between the number of ROS produced and the antioxidant system causes neural disorders. Although there is no unified etiology for neurodegenerative diseases, strong evidence has implicated oxidative stress in the pathogenesis of all forms of neurodegeneration [6, 7]. Overproduction of reactive oxygen species in neuronal cells induces oxidative stress, which destroys neural mitochondrial defense mechanisms, causes mitochondrial DNA mutations, affects Ca^2+^ homeostasis, alters membrane permeability, and damages the mitochondrial respiratory chain. These modifications are thought to play a role in the progression of neurodegenerative disorders by mediating or amplifying neuronal dysfunction and causing neurodegeneration [5].

A series of events, such as oxidative stress, protein modification, and mtDNA damage, eventually results in impaired neuronal proteins, further resulting in neuroinflammation and neurological disorders, which manifest as cognitive function loss [8]. However, numerous antioxidant therapeutic targets have been identified that can protect neurons against oxidative stress by preventing free radical formation and modulating normal metal homeostasis [7]. Antioxidants are now being developed to treat neuronal inflammation and scavenge free radicals. The disruption of the redox potential in neurons often results in the oxidative modification of proteins, leading to aberrant peptide oligomerization and eventually neuronal death. Research efforts have focused on discovering agents that can protect against oxidative damage to neurons [9]. In this paper, we reviewed scientific reports on the pathogenesis and prospects for therapeutically targeting oxidative stress in neurodegenerative diseases, especially Alzheimer's and Parkinson's disease.

## 2. Reactive Oxygen Species and Oxidative Stress

**Figure 1:**
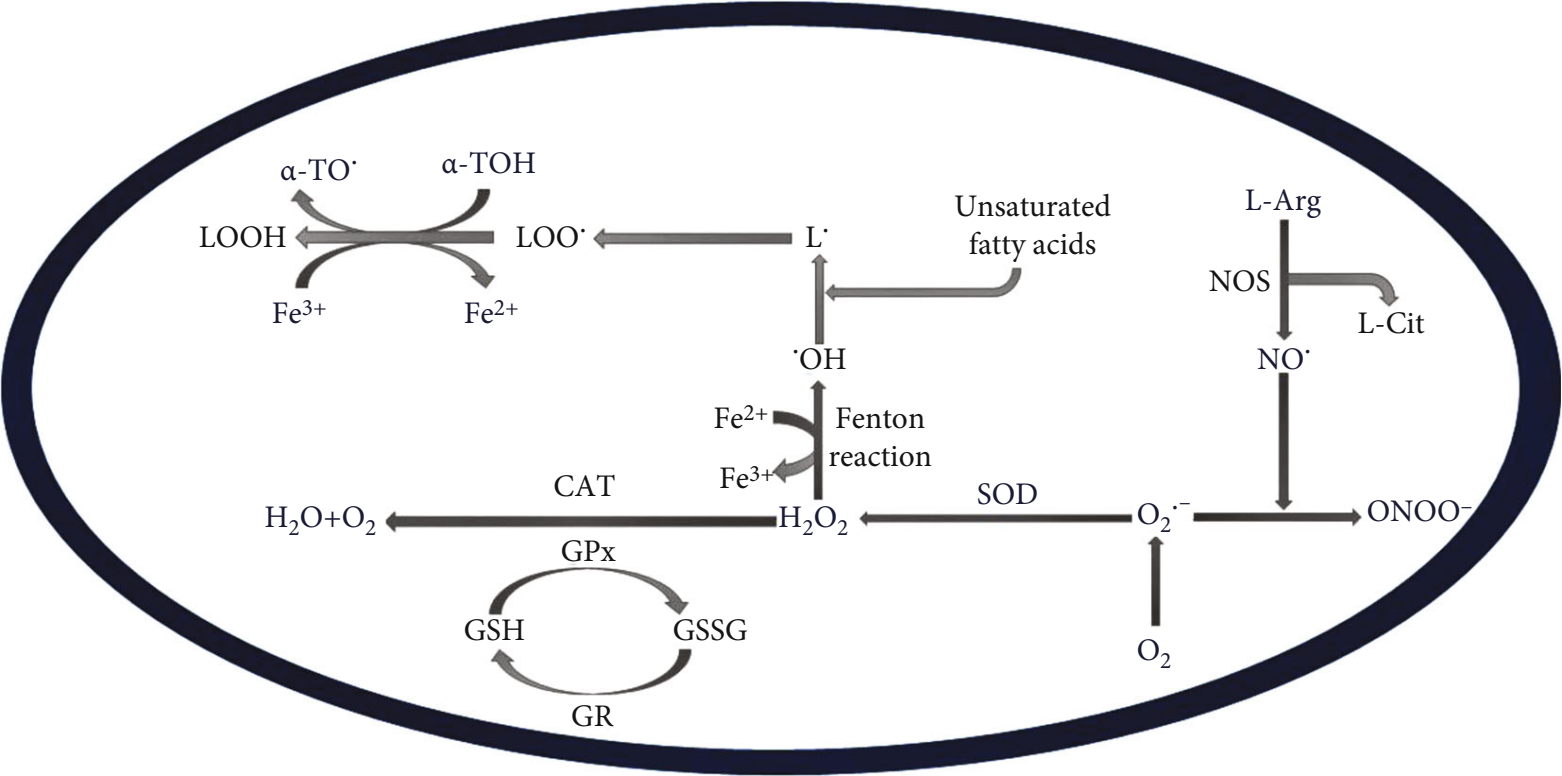
Generation of reactive oxygen species and reactive nitrogen species. H_2_O: water; O_2_: oxygen; O_2_^·-^: superoxide anion; H_2_O_2_: hydrogen peroxide; CAT: catalase; SOD: superoxide dismutase; ONOO^−^: peroxynitrite; GSH: reduced glutathione; GSSG: glutathione disulfide; Fe^2+^: ferrous iron; ^·^OH: hydroxyl radical; NOS: nitric oxide synthase; L-Arg: L-arginine; L-Cit: L-citrulline; NO^·^: nitric oxide radical; LOOH: lipid hydroperoxides; LOO^·^: lipid peroxy radical; *α*-TOH: *α*-tocopherol; *α*-TO^·^: *α*-tocopheroxyl radical.

Oxidative stress arises as a result of an imbalance in the oxidant/antioxidant ratio and can create a hazardous state that contributes to cellular damage due to a disequilibrium in the number of ROS molecules generated and the level of the antioxidant enzyme system that detoxifies the reactive intermediates in the biological system [10]. The majority of free radicals (hydrogen peroxide, superoxide anion radical, and hydroxyl radical) are produced by molecular oxygen activation (Figure 1). Superoxide anion (O_2_^·-^) is highly reactive and generated in the electron transport chain (ETC) through mitochondrial complexes I and III. O_2_^·-^ can be reduced to hydrogen peroxide (H_2_O_2_) as it crosses the inner mitochondrial membrane [11]. Peroxisomes can also generate H_2_O_2_ in addition to what is produced by the mitochondria. However, catalase is present in the peroxisomes and is responsible for converting H_2_O_2_ to water, thereby preventing its buildup. However, if there is damage to the peroxisomes or the enzyme is downregulated, the release of H_2_O_2_ into the cytosol increases oxidative stress [12]. Direct pathways involving Fenton and Haber-Weiss reactions that occur between redox-active metals and oxygen species or indirect interactions involving enzyme activation (NADPH oxidase or nitric oxide synthase) are examples of pathways that generate ROS [13, 14]. The Fenton reaction in the presence of ferrous iron (Fe^2+^) converts H_2_O_2_ into the most harmful ROS, the hydroxyl radical (^·^OH) (Figure 1) [13].

ROS can also interact with nitric oxide to generate reactive nitrogen species resulting in nitrosative stress. NO is produced by one of the three synthase isoforms, inducible NO synthase (iNOS), endothelial NO synthase (eNOS), and neuronal NO synthase (nNOS), and is found in cells and in the extracellular space surrounding dopaminergic neurons. nNOS is expressed mainly in neurons, eNOS is expressed in the vascular endothelium, and iNOS is expressed in glial cells. These NOSs have been implicated in pathological conditions. Damaged mitochondria and activated microglia produce large amounts of iNOS and serve as the main sources of ROS in the inflammatory process, which is a hallmark of neurodegenerative diseases [15–18]. For instance, the production of NO by iNOS or nNOS in the extracellular space surrounding dopaminergic neurons and its subsequent reaction could involve interaction with superoxide to produce large quantities of peroxynitrite (ONOO^−^) (Figure 1) [19]. Compared to NO, peroxynitrite is a more potent oxidizing agent and is an oxidatively more active molecule which can induce lipid peroxidation and DNA fragmentation [20]. The generated ONOO^·^ and NO can also modify proteins via nitration [21]. NO can also react with thiolate ions to form S-nitrosothiols, which may form adducts with protein or lipids. Another cellular mechanism implicated in neurodegenerative conditions that involve the production of ROS occurs in the endoplasmic reticulum (ER). Ca^2+^ release from the ER leads to the promotion of ROS generation when the Ca^2+^ enters the mitochondria and can also trigger mitochondrial-mediated apoptosis. Additionally, abnormal protein degradation and ER stress are considered to contribute to PD pathogenesis [22].

## 3. Neuronal ROS Generation

Cellular ROS can be generated via exogenous and endogenous sources. Exogenous sources include metabolism of xenobiotics and exposure to ultraviolet and ionizing radiation, whereas endogenous generation is mediated by enzymes such as NADH, coenzyme Q oxidoreductase (complex 1), ubiquinol cytochrome C reductase (complex III), NADPH oxidase (NOX), lipoxygenase (LOX), cyclooxygenase (COX), xanthine oxidase (XO), the cytochrome p450 family of enzymes, and flavin oxidase. The majority of these enzymes are localized in the mitochondria, and as such, the mitochondria are the primary source for the endogenous generation of ROS [23].

Neurons are energy-demanding cells; as such, they are extremely sensitive to changes in mitochondrial function. They depend on the mitochondria for ATP generation and calcium regulation, processes required for synaptic transmission, vesicle recycling, neuronal electrical activity, and axonal transport [6]. Thus, deficiencies in mitochondrial function ultimately result in the degradation of neurons. Neurodegeneration is an age-related disease; it is associated with mitochondria, DNA damage, mutations, and deletions, which increase in an age-dependent manner. Based on evidence, the levels of mutated or deleted mtDNA are the highest in the substantia nigra (SN) and cerebral cortex (CC) of adult humans in normal aging, and these damages are often 1% higher in patients with neurodegenerative diseases [24]. The neurons in the brain regions of subjects with neurodegenerative diseases often show alterations in the ETC, intracellular ROS levels, and calcium homeostasis. The processes of mitochondrial fusion and fission are deregulated in most neurodegenerative diseases, leading to impaired neuronal plasticity and development [6].

Mitochondrial dysfunction and the increased generation of ROS alter dopamine metabolism and calcium and iron homeostasis, resulting in the death of dopaminergic neurons in the substantia nigra pars compacta (SNpc). The eventual depletion of antioxidants such as glutathione (GSH) also leads to the generation and accumulation of lipid peroxides as ROS interacts with abundant polyunsaturated fatty acids (PUFA) in the brain [25–27]. Reports also show that aging is accompanied by an increased level of iron and copper in the brain tissue; such alterations in ion homeostasis could initiate excitotoxicity via the overactivation of neuronal receptors such as N-methyl-D-aspartate (NMDA) receptors [28]. The aberrant activation of NMDA receptors produces high levels of NO, which could interact with superoxide and induce nitrosative stress [29].

## 4. Sources of ROS Generation in Neurons

### 4.1. Iron

Ions such as iron and calcium are involved in the generation of ROS. Postmortem evaluation of the brains of patients with ND (neuronal disease) indicates that alterations in the levels of certain ions are present in the specific brain regions of ND patients compared with non-ND [30]. Iron is an indispensable cofactor for the synthesis of catecholamine neurotransmitters, but neuronal iron overload is a characteristic feature of neurodegenerative diseases [31]. In dysfunctional neurons, iron is transported from the extracellular matrix around the neurons via mechanisms such as transport via transferrin lactoferrin and hemopexin receptors and divalent metal transporter 1 as well as by heme degradation by heme oxygenase 2 [31–33]. Since iron is abundant in dysfunctional neurons, free iron interacts with the reaction between superoxide and hydrogen peroxide with ferrous iron (Fe^2+^) and ferric iron (Fe^3+^) resulting in the formation of a highly reactive hydroxyl free radical. Neuronal iron overload in ND is often accompanied by depleted iron concentration in the extracellular matrix; this results in poor iron incorporation into protein structures, premature protein degradation, and loss of protein function [31, 34–36]. Lewy's SN bodies of PD patients are characterized by nitrosylated iron-regulated protein 2 (IRP), which promotes the possibility of oxidative stress and iron dysfunction [37].

The human brain is heavily endowed with PUFAs, such as docosahexaenoic acid and arachidonic acid, which are required to fortify the blood-brain barrier (BBB). This makes the cell membrane of brain tissues susceptible to lipid peroxidation and oxidative damage [38]. Interaction between iron and lipid hydroperoxides results in the production of alkoxyl radicals, which further react in the presence of free reactive iron with polyunsaturated fatty acids to generate more lipid peroxides. 4-Hydroxy-2-nonenal (HNE), which is a lipid peroxide associated with PD and AD, forms stable adducts with amine or thiol groups in proteins and may, in turn, result in the aberrant activation of caspases, leading to neuronal death [39, 40]. HNE reactivity with sulfhydryl groups also depletes the GSH level, making the brain tissue more susceptible to oxidative stress. High HNE levels have been detected in the cerebrospinal fluid of patients with PD and AD [41].

### 4.2. Zinc and ROS Generation

Experiment carried out on mouse cortical cultures showed mitochondrial ROS production induced by zinc, possibly via inhibition of the lipoamide dehydrogenase (LADH) component of alpha-ketoglutarate dehydrogenase complex (KGDHC) in the TCA cycle [42, 43]. LADH catalyses NAD^+^ reduction to NADH, but zinc inhibits this process and favours NADH oxidation in the mitochondrial matrix. This reaction produces superoxide, which is converted to H_2_O_2_. Complex III inhibition by zinc also generates ROS in rat brain mitochondria which is also similar to that reported in mitochondria isolated from rat hearts [44, 45]. ROS generation via zinc may be a result of its inhibition of ETC components and TCA cycle. Some other sources of zinc-induced ROS generation include nicotinamide adenine dinucleotide phosphate (NAPDH) oxidase (NOX) upregulation. NOX converts NADPH and oxygen to NADP+, superoxide and a hydrogen ion [46, 47].

### 4.3. Aluminium and ROS Generation

Aluminium (Al) generates highly reactive oxy and hydroxy free radicals which result in mitochondrial damage. Al increases oxidative stress via mediated Fenton reactions which increase the concentration of iron and it can also lead to H_2_O_2_ accumulation [48]. Al also activates SOD and inhibits CAT. Increase in H_2_O_2_ pool increases redox-active iron present either by modulating the electron transport chain or from loosely bound Fe. Studies have shown that oxidative stress and A*β* are closely linked and Al increases the production of A*β*, leading to aggregation [49]. Another study also revealed that Al initially enhances oxidative stress, redox-active iron, and A*β* immunoreactivity [50, 51]. The Fenton reaction helps Al to cause a lot of havoc via increasing the redox active iron concentration in the brain. Thus, Al significantly plays a role in neurodegeneration through oxidative stress.

## 5. Parkinson's Disease and Oxidative Stress

Parkinson's disease (PD) is a major neurological disease common among the aged (>60 years); it is characterized by a progressive loss of motor control [52]. PD has become the second most reported neurodegenerative disease with around 10 million people having the condition worldwide [53]. PD is more predominant among men than women and seems to be strongly attributable to exposure to environmental toxins, illness, and head trauma [54]. Some of the clinical features of PD are impaired balance, tremor, rigidity, and bradykinesia, which progress to cognitive, sensory, psychiatric, and autonomic impairments [55]. Other manifestations of PD include memory loss, depression, anxiety, insecurity, stress, constipation, diminished sense of smell, difficulty in swallowing and excessive salivation, skin problems, confusion, erectile dysfunction, increased sweating, and a monotone voice [56].

**Figure 2:**
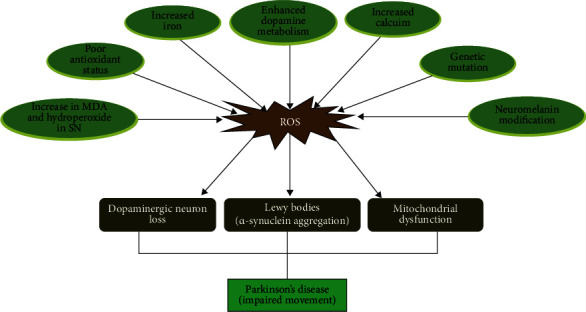
The role of oxidative stress in the pathology of Parkinson's disease.

The main risk factor for Parkinson's disease is age, with the disease's occurrence increasing exponentially after 60 years. Apart from the years it takes for pathogenic proteins to misfold to the point where they cause neuronal damage, age-related mitochondrial dysfunction and high generation of ROS are vital factors in neurodegenerative disorders [57]. Elevated deletions in mtDNA are reported in SN pigmented neurons in aged and PD-affected brains [58]. mtDNA damage results in the expression of mutant forms of ETC subunits and mitochondrial tRNAs, both of which contribute to aberrant ROS production, leading to a vicious cycle of mtDNA damage and other mitochondrial dysfunction, which also increase the development of ROS (Figure 2).

Sustained exposure to pesticides and insecticides has been identified as a risk factor for PD. According to Marras et al. [54], higher levels of pesticides and organochlorine insecticides and their metabolites can be detected in the serum and SN of PD patients than of non-PD subjects. Silver et al. [59] also suggested that the utilization of well-water contaminated with pesticides could increase the PD risk. Paraquat and rotenone, active ingredients in herbicides and pesticides, have been linked to PD-associated oxidative stress upon entry into a biological system; they are metabolized to generate reactive species, which could damage mtDNA and inhibit enzymes involved in the ETC [60–62].

The pathogenesis of PD has shown that the disease involves the gradual and selective degradation of neurons in the SN, an area responsible for movement. The SN contains numerous neurons that release dopamine and then communicate with both the basal ganglia and the frontal lobe responsible for the movement [63]. The oxidation of dopamine to produce dopamine quinones results in a generation of a highly reactive aminochrome that generates superoxides and depletes NADPH pools [64]. Aminochrome in turn forms an adduct with *α*-synuclein, and this stimulates disease progression. Postmortem brain analysis in the SN of PD patients has revealed a significant increase in dopamine cysteinyl adducts [65, 66]. Dopamine cysteinyl adducts become a precursor for the synthesis of neuromelanin, a molecule responsible for inflammation and degeneration in catecholaminergic neurons (Figure 2) [65, 67].

Postmortem examination of the SN brain regions of PD patients indicates a significant reduction in reduced glutathione (GSH) levels relative to glutathione disulfide (GSSG) level ratio (GSH/GSSG ratio) compared to non-PD patients [68]. The decreased GSH levels could be indicative of glutathione reductase inhibition in the SN of PD subjects as studies have shown that progressive nigral dopaminergic neurodegeneration leads to a downregulation of GSH production [25, 69].

Interestingly, mitochondrial anomalies have also long been considered as a major factor underlying PD pathogenesis, and studies have revealed the huge part played by mitochondrial dynamics and quality control in PD. In brief, implications of the involvement of mitochondrial dysfunction in PD stem from findings that (1) substantia nigra neurons from PD patients exhibit accumulations of mtDNA deletions; (2) significant deficits occur in the activity of mitochondrial respiratory chain complex I; and mutants of PINK-1, DJ-1, and Parkin, which are involved in mitochondrial functioning [70, 71], are found in PD.

## 6. Alzheimer's Disease and Oxidative Stress

Alzheimer's disease (AD) is an irreversible neurodegenerative disease, characterized by neuronal degradation in brain regions that control cognitive, memory, and emotional functions [72]. AD is age-related, progressive in nature, and can only be managed but not cured. The global prevalence of AD is estimated to rise from 26 million cases to 100 million cases by 2050 [73, 74].

The hallmarks of Alzheimer's disease are the formation of neurofibrillary tangles and senile plaques, neuronal and synaptic loss, severe neuroinflammation, and a global decrease in brain volume [72, 75]. Although the complex nature of AD makes it difficult to accurately diagnose in living patients, advances in biomedical engineering have provided a wide array of tools that can diagnose AD in living patients with at least 95% accuracy. These tools include clinical tests to rule out other disease states, such as deficiencies in certain vitamins, infections, cancers, depression; neuropsychological tests for the evaluation of cognitive function; the use of cerebrospinal fluid protein analysis to differentiate AD from other dementias, especially, Creutzfeldt-Jakob disease, Lewy body dementia, or frontotemporal dementia; magnetic resonance imaging to check for shrinkage of brain regions responsible for learning and memory; and the detection of abnormal protein deposits using positron emission tomography scans [74, 76].

The etiology of AD is sporadic, and there is no single generally accepted cause of AD. However, about 5% of known cases are caused by a germline mutation in presenilin 1 or 2 (PSEN 1 or 2) and/or amyloid precursor protein (APP) [77]. After translation of the APP gene to about 11 different isoforms, the protein undergoes amyloidogenic or nonamyloidogenic posttranslational modifications into various proteins, one of which is the amyloid-*β* (A*β*) protein, a prominent culprit in the pathology and progression of AD [78]. Mutations in the APP gene produce misfolded A*β* peptides/proteins. The resultant formation of senile plaques fosters neurofibrillary tangle synthesis via tau protein hyperphosphorylation, a second protein culprit in the pathogenesis of AD [79, 80]. The neurofibrillary tangles and senile plaque accumulation may occur decades before cognitive and memory deficits are observed in AD patients [81].

**Figure 3:**
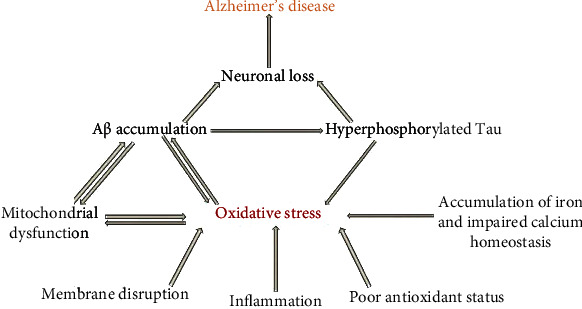
The role of oxidative stress in Alzheimer's disease.

Mitochondrial morphology has become a major factor in AD pathogenesis. For instance, compared with age-matched controls, AD neurons contain a large number of mitochondria with broken cristae. Also, the size and number of the neuronal mitochondria, especially those of the CA1/CA3 regions of the hippocampus, dentate gyrus, and the entorhinal cortex, differ from those in normal participants [82, 83]. Furthermore, fibroblasts from AD patients demonstrate abnormal mitochondrial dynamics [82]. The overall expression levels of the fission/fusion proteins DLP-1, OPA-1, Mfn1 and 2, and Fis1 are altered in AD (Figure 3).

Extensive research on the pathology of AD indicates that A*β* hastens the synthesis of neurofibrillary tangles via the activation of specific kinases involved in tau protein phosphorylation [84, 85]. Normal tau proteins are “natively unfolded” and have an extremely low tendency towards misfolding, aggregation, and accumulation (Figure 3). They are involved in microtubule assembly and stability. Microtubules are the structural and functional backbone of neurons and are involved in intracellular trafficking and transport and in the maintenance of cell polarity and microtubules [86, 87], so normal tau proteins are also indirectly involved in these roles. Upon hyperphosphorylation, tau proteins lose their charge and conformation, which causes them to self-aggregate and oligomerize [88, 89]. These aggregates are eventually converted to intracellular neurofibrillary tangles, which result in microtubule disassembly, leading to the cessation of intracellular transduction and to neuroinflammation and neuronal degradation [90, 91].

AD progresses in three stages. First, there is the gradual buildup of extracellular senile plaques and intracellular neurofibrillary tangles, followed by mild cognitive impairment (MCI), which is linked with the buildup of plaques and tangles, and ending with early AD [72]. Several *in vitro* studies suggest that mitochondrial dysfunctions resulting from the production of reactive oxygen species (ROS) and reactive nitrogen species (RNS) [72, 92–94] play a detrimental role in the progression of AD. Oxidative-associated modifications in membrane-associated macromolecules and nucleic acids have been noted in the brains of AD subjects [95]. According to Haluska et al. [96], increased levels of lipid peroxides and aldehyde-4-hydroxynonenal (HNE) were detected in the brain during the early stages of AD models. Oxidation of nuclear and mitochondrial DNA/RNA molecules, especially of guanosine bases to 8-hydroxyguanosine, in brain regions that coordinate cognitive and emotional functions has also been documented [95].

Redox proteomics is essential for identifying specific proteins that are differentially oxidized in specific brain regions of AD patients. Specifically, several analyses have shown that the levels of protein nitration, protein carbonyls, and 4-hydroxynonenal-modified proteins increase significantly in the brains of MCI AD patients [97, 98]. The identified oxidatively modified proteins involved in regulating energy metabolism, cellular communication, and tau hyperphosphorylation include phosphoglycerate, enolase 1, pyruvate kinase, lactate dehydrogenase, peroxiredoxin, creatine kinase B, glucose-regulated protein precursor, glutamine synthetase, actin, *α*-tubulin, neuropolypeptide, and prolyl isomerase [72].

AD-associated oxidative stress is mediated by A*β*, which fosters disease progression. For instance, Giraldo et al. [99] stated that A*β*-mediated oxidative stress triggers the oxidation of both NADPH oxidase and p38; the aberrant activation of p38, in turn, triggers the hyperphosphorylation of tau proteins. A*β*-mediated oxidative stress also plays an important role in the activation of calcineurin, sometimes known as a death molecule, which activates Bcl-2 death-associated proteins and fosters cytochrome c release from the mitochondria resulting in the apoptotic death of neurons [100]. A*β* deposits contain elevated levels of copper, zinc, and iron ions, which could interact with A*β* to produce ROS via the Fenton reaction [101]. These ions could also bind to tyrosine and histidine residues of A*β*, resulting in the oligomerization of A*β* proteins to form senile plaques [102, 103].

## 7. Therapeutic Intervention in Parkinson and Alzheimer's Disease

Over the last few years, intense study on multiple fronts has advanced the understanding of the genetics and mechanisms of neuronal pathogenesis and has contributed greatly to our understanding of neurodegenerative diseases, creating a basis for innovative technologies and therapeutic interventions against neurodegenerative diseases [4]. However, there are no available drugs for delaying the progression of PD or AD although some drugs have been approved for the relief of PD and AD symptoms. Available therapeutic agents for PD often work by inhibiting striatal cholinergic effects or enhancing the activity of dopaminergic neurons [104]. Inhibitors of N-methyl-D-aspartic acid (NMDA), such as memantine and acetylcholinesterase (AChE), which include donepezil, rivastigmine, and galantamine, are also currently used to alleviate AD-related symptoms. Although these agents have succeeded in stimulating mild improvements in cognitive and memory functions, they do not reverse or delay PD and AD progression [101]. Since oxidative stress has been implicated in the pathology and progression of AD, current research trends are shifting focus to the use of antioxidative agents in PD and AD treatment.

**Figure 4:**
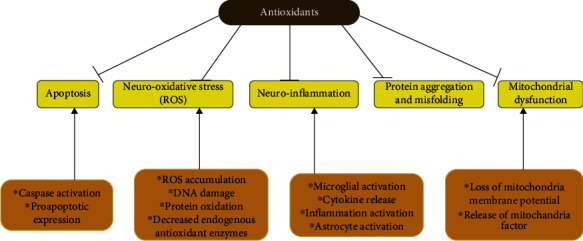
Antioxidant intervention at the cellular and molecular levels in neurodegeneration.

**Figure 5:**
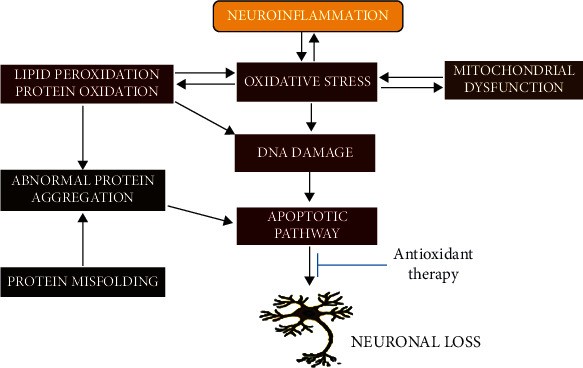
Schematic diagram on the effects of antioxidant on neuronal damage.

The molecules that have displayed great antioxidant activity when treating neurodegenerative diseases in *in vitro* and *in vivo* models are present in pure natural products or plant extracts. However, clinical outcomes in human patients have had limited success and are still inconclusive. This could be linked to the use of single compounds in most clinical trials. In contrast, preclinical studies have used plant extracts that contain several secondary metabolites. The synergistic effects of several active ingredients may provide better antioxidant/disease-modifying activity (Figures 4 and 5) [105–108].

## 8. Targeting Oxidative Stress in Parkinson's Disease

Antioxidants protected against PD pathogenesis in several in vitro and in vivo studies. However, the defense was only partial, leaving an open window for identifying more appropriate antioxidants for use as PD therapeutics [9]. Endogenous antioxidants, such as coenzyme Q10 (CoQ10), protect cells from oxidative stress (Figure 4). Coenzyme Q10, which is expressed in the mitochondria, acts as a free radical scavenger and electron carrier and accepts electrons released by complexes I and II. Coenzyme Q10 functions as a cofactor and may reduce free radical formation by activating the mitochondrial uncoupling protein. CoQ10 and creatine have shown great promise and are undergoing clinical trials for Parkinson's disease [109, 110].CoQ10 is involved in *α*-tocopheroxyl radical reduction and the regeneration of *α*-tocopherol, making it a powerful free radical scavenger. CoQ10, which reduces the generation of free radicals and regulates the production of ATP, is also a required cofactor for mitochondrial uncoupling proteins [111].

Curiously, CoQ10 levels are reduced in platelets of PD patients, and this may correspond to mitochondrial complex I activity deficiency [112]. CoQ10 has also been shown to prevent death from oxidative stress, apoptosis, and cell death by inhibiting the mitochondrial permeability transfer pore (MPTP), which causes cell death by increasing mitochondrial calcium retention. Because of the decreased level of CoQ10 in PD patients, ATP synthesis is altered and mitochondrial membrane disruption occurs. Significantly improved mitochondrial dysfunction, depletion of dopamine, and axonal degeneration of dopamine neurons following oral CoQ10 were also found in experimental models of PD and in PD patients [113]. CoQ10's underlying protective mechanism is still not clearly defined. CoQ10 may reduce apoptosis by decreasing the Bl-2 antiapoptotic factor level, inhibits cytochrome c release to maintain the integrity of the membrane, and stops caspase 3 and caspase 9 activation to protect cells against apoptosis [96]. Other naturally occurring cellular molecules, such as creatine, have shown antioxidative properties [114].

The use of creatine in PD treatment is not well established, so further investigation is required. Exogenous polyphenolic compounds such as curcumin, resveratrol, quercetin, puerarin, luteolin, genistein, epigallocatechin gallate, baicalein, and citrus flavonoids (naringenin and hesperidin) can cross the blood-brain barrier and may be neuroprotective during the progression of Parkinson's disease. Extensive research has shown that diets supplemented with polyphenols are beneficial to health. Polyphenols provide neuroprotection against a variety of neuropathological and neurodegenerative conditions [115, 116].

Studies of the pathways of the Nrf2/antioxidant response element (ARE) indicate that it protects neurons from the PD insult of oxidative stress (OS). Two novel drug categories involving this signaling pathway have been developed. These are pathologically activated therapeutics and proelectrophilic drugs, both of which activate the Nrf2/ARE pathway through powerful cellular defense systems against OS [117]. In SH-SY5Y cell models of PD, high Nrf2 protein levels and gene activation of the ARE pathway were induced by naringenin, a natural flavonoid compound [118]. Lou and colleagues demonstrated that 6-OHDA-induced oxidation in SH-SY5Y cells was ameliorated by naringenin via Nrf2/ARE signaling [118]. Additionally, an antioxidant effect of a small catechol, 3,4-dihydroxybenzalacetone, is also involved in the AKT/Nrf2/glutathione pathway [119]. Wei et al. found that Wnt3a could reduce 6-OHDA-induced neurotoxicity through the activation of the Wnt/b-catenin pathway, implying that the mechanism underlying OS inhibition is related to mitochondrial functional maintenance [120].

Moreover, several antioxidants have been studied to determine if they can improve the damage caused by OS in Parkinson's disease. For example, behavioral studies indicated that sulforaphane reduced 6-OHDA-induced rotation and motor coordination deficits in mice [121]. Standardized extracts of *Hypericum perforatum*, gallic acid, and *Sida cordifolia* had potent antioxidant activities in a 6-OHDA rotenone-induced rat model of Parkinson's disease [122]. Several drugs, heavy metals, carotenoids, oxathiolene oxides, dimercaptans, dithiocarbamates, trivalent arsenicals, 1,2-dithiole-3-thiones, isothiocyanates, and quinones, have been reported to interact with specific redox-sensitive cysteines in Keap1 to dissociate Nrf2 from Keap1. There is strong evidence that these Nrf2 inducers may help slow down PD progression [123]. Similar results have been reported for dietary polyphenols, such as resveratrol, curcumin, or certain catechols. In addition, dopamine agonist (DA) and its derivatives as well as levodopa may hold prospects for PD treatment. These compounds are not electrophilic, but become electrophilic by oxidative conversion, which leads to increased expression and activity of GSK-3*β* (glycogen synthase kinase–3 beta). Besides aging, the major risk factor for PD has been identified as being a steady increase in GSK-3*β* activity and a decline in Nrf2 transcriptional activity. GSK-3*β* inhibitors including antioxidative phytochemicals have been shown to protect against the death of oxidative neuronal cells induced by stress [124, 125]. Overall, phytochemicals appear to be a promising treatment option for current neurodegenerative diseases.

Finally, understanding the link between oxidative stress and impaired mitochondrial function could indicate that targeting oxidative stress by using antioxidants may also confer protection for mitochondria. Preclinical studies have shown that mitochondria-targeted antioxidants (such as CoQ10, vitamin E, MitoQ, or urate) improve mitochondrial activity, but creatine, MitoQ, and coenzyme Q10 did not demonstrate disease-modifying benefits in PD patients in clinical trials. Taking these results together indicates that oxidative stress is a downstream effect of mitochondrial dysfunction rather than a direct cause of PD-related neurodegeneration [126–129].

## 9. Targeting Oxidative Stress in Alzheimer's Disease

Some experiments in transgenic mouse models and some human clinical trials demonstrated that *α*-tocopherol- and selenium-supplemented diets promote the proteolytic degradation of A*β* plaques, ameliorate the aggregation of tau proteins, and improve cognitive function [130–133]. These findings were, however, contradicted by a 5-year clinical trial that revealed that the supplemental use of *α*-tocopherol and selenium only treated AD symptoms but did not prevent the progression of AD [134]. According to Yang et al. [135], the little or no improvement in cognitive function in response to antioxidant supplements during clinical trials could have resulted from ineffective delivery of the antioxidant to the mitochondria of neurons. With that in mind, some researchers now focus on the targeted delivery of antioxidants to the mitochondria of neurons. Gao et al. [136] reported that the biomimetic nanosystem designed for their study effectively crossed the blood-brain barrier and delivered curcumin to the mitochondria of neurons. Based on the findings reported by Yang et al. [135], the resveratrol-encapsulated neuronal mitochondria-targeted micelle designed for their study was able to facilitate the delivery of high levels of resveratrol into the brain mitochondria; this efficiently reduced oxidative stress, decreased A*β* plaque formation, and ameliorated tau hyperphosphorylation, neuroinflammation, and declined memory function in an aged AD mice model.

Another strategy that has been adopted for targeting oxidative stress in AD patients involves the use of AChE inhibitors with strong antioxidant potential and good blood-brain barrier-permeating abilities. According to Yang et al. [101], a series of newly synthesized 8-hydroxyquinoline derivatives with good brain barrier permeating ability, strong antioxidative activity, and significant metal-chelating ability was able to inhibit the generation of A*β* fibrils and aggregates in AD mouse models. Srivastava et al. [137] also reported that a new class of N-(4-phenoxybenzyl) aniline derivatives designed for their study improved learning and memory functions in transgenic mice via mechanisms that involve the inhibition of acetylcholinesterase, the formation of A*β* aggregates, and the reduction of oxidative stress.

Recently, the pharmacologically developed compound diethyl(3,4-dihydroxyphenethylamino) (quinolin-4-yl) methylphosphonate (DDQ) demonstrated positive effects on mRNA and protein levels related to mitochondrial dysfunction and synaptic dysregulation. DDQ also had an effect on mitochondrial dynamics related to A*β* interactions, fusion proteins (Mfn1 and 2), and fission proteins (Lis1 and DRP1) [138]. The table below shows a summary of natural antioxidative compounds that have been reported for therapeutic effects against AD and PD (Table 1).

## 10. Future Directions

The intervention of a single or few antioxidants to treat neuronal dysfunction may be termed as too simple to combat the complex ROS metabolism due to their results seen in clinical trials. An effective approach to antioxidant therapy would be to decrease ROS generation and upregulate the various intracellular and mitochondrial antioxidant defense system. For example, Nrf2 activates the antioxidant enzyme network and is termed as the master regulator of redox homeostasis. Therefore, therapeutic target of Nrf2 in the neuron offers a reliable means of regulating oxidative stress. Although, however, controlling oxidative stress alone may not be enough to target neurodegenerative diseases, the restoration of the neuronal function may depend also on targeting some other cell survival pathways. A considerable understanding of ROS biology will also provide insights into what can be targeted than what we have at present.

## 11. Conclusion

The underlying mechanisms of neurodegenerative disorders are not fully understood; however, there is strong evidence that redox imbalance not only plays a key role but mostly precedes neurodegenerative manifestations. Redox control in physiologic processes is important for maintaining the redox balance and for achieving redox homeostasis. Cells counteract redox imbalance by harnessing an array of both endogenous and exogenous redox-active substances through multiple cellular processes or pathways. Identifying and targeting these redox-regulated pathways in neurodegeneration may provide an avenue for newer treatment strategies. Moreover, as our understanding of the role of redox-regulated events in neurodegeneration becomes clearer, compounds such as phytochemicals that have antioxidative, anti-inflammatory, and anticholinesterase properties may be explored as a source of new treatments for neurodegenerative disorders in the future.

## Figures and Tables

**Table 1:** Natural compounds with therapeutic action against neurodegeneration disorders.

Compounds	Therapeutic target	Mechanism of action	Reference
Coenzyme q10	PD	*α*-Tocopheroxyl radical reduction and regeneration of *α*-tocopherol Suppression of nigrostriatal dopaminergic cell death	[9, 110, 113]
Luteolin	PD	Increases dopamine uptake	[139]
Resveratrol	PD, AD	Nrf2 activation	[115, 116, 123, 135]
Curcumin	PD	Nrf2 activation	[123]
Epigallocatechin gallate	PD	Nrf2 activation	[123]
*α*-Tocopherol	AD	Proteolytic degradation of A*β* plaques	[130–133]
Quercetin	PD	Scavenges hydroxyl radicals	[140]
Selenium	AD	Proteolytic degradation of A*β* plaques	[130–133]
8-Hydroxyquinoline derivative	AD	Inhibit A*β* fibril formation and aggregation	[82]
N-(4-Phenoxybenzyl) aniline derivative	AD	Inhibition of acetylcholinesterase	[137]

## Data Availability

All data are included in the manuscript.

## Abbreviations

ND: Neurodegenerative disease
PD: Parkinson's disease
AD: Alzheimer's disease
EAD: Early Alzheimer's disease
MCI: Mild cognitive impairment
SNpc: Substantia nigra pars compacta
BBB: Blood-brain barrier
SN: Substantia nigra
CC: Cerebral cortex
mtDNA: Mitochondrial DNA
nNOS: Neuronal nitric oxide synthase
eNOS: Endothelial nitric oxide synthase
iNOS: Inducible nitric oxide synthase
NADPH: Nicotinamide adenine dinucleotide phosphate
NOX: NADPH oxidase
LOX: Lipoxygenase
COX: Cyclooxygenase
XO: Xanthine oxidase
H_2_O_2_: Hydrogen peroxide
O_2_^·-^: Superoxide anion
ETC: Electron transport chain
^·^OH: Hydroxyl radical
GSH: Glutathione
NMDA: N-Methyl-D-aspartate
HNE: Hydroxynonenal
AChE: Acetylcholinesterase
CoQ10: Coenzyme Q10.
